# pH-Resolved ATP Synthesis in Skeletal Muscle: Concept, Implementation, and Assessment Using Dynamic ^31^P Magnetic Resonance Spectroscopy at 7T

**DOI:** 10.3390/diagnostics16050744

**Published:** 2026-03-02

**Authors:** Jimin Ren, Neha Patel, Ross Querry, Staci Shearin, Jarett Berry, Wanpen Vongpatanasin

**Affiliations:** 1 Advanced Imaging Research Center, University of Texas Southwestern Medical Center, Dallas, TX 75390, USA; 2 Department of Radiology, University of Texas Southwestern Medical Center, Dallas, TX 75390, USA; 3 Department of Physical Therapy, University of Texas Southwestern Medical Center, Dallas, TX 75390, USA; 4 Department of Internal Medicine, University of Texas Southwestern Medical Center, Dallas, TX 75390, USA; 5 Department of Internal Medicine, University of Texas at Tyler School of Medicine, Tyler, TX 75799, USA

**Keywords:** pH, skeletal muscle, ATP synthesis, mitochondrial dysfunction, metabolism, exercise, aging

## Abstract

**Background/Objectives:** Dynamic changes in inorganic phosphate (Pi), phosphocreatine (PCr), and pH during post-exercise recovery reflect underlying muscle energetics and mitochondrial ATP synthesis, but the conventional single-pool model assuming uniform metabolic response fails to address myofiber composition and pH-dependent metabolic heterogeneity in skeletal muscle. This study aimed to characterize the interplay between pH, Pi, and PCr, and to develop an analytical method for assessing pH-resolved ATP synthesis using ^31^P MRS. **Methods:** Five healthy subjects underwent dynamic ^31^P MRS scans during plantar flexion exercise. ATP synthesis was evaluated from post-exercise PCr and Pi recovery time courses using the single-pool model, and from Pi recovery time courses using a multi-pool model in which the Pi signal lineshape was segmented into four pH-specific pools: alkaline (pH 7.3 ± 0.2), neutral (pH 7.0 ± 0.1), mildly acidic (pH 6.8 ± 0.1), and moderately acidic (pH 6.6 ± 0.1). **Results:** The single-pool model showed that during exercise, Pi increased proportionally to PCr depletion, and both Pi and PCr recovered monoexponentially immediately after exercise with $\tau_{\mathrm{Pi}}\;\left(33\pm 9\;s\right)<\tau_{\mathrm{PCr}}\;(40\;\pm 9\;s)$; ATP remained stable while pH exhibited a “heart-beat” pattern, characterized by an initial alkalization followed by neutralization during exercise, a post-exercise acidic undershoot, and a subsequent slow recovery ($\tau_{\mathrm{pH}}\gg \tau_{\mathrm{PCr}}$). The four-pool model demonstrated a pronounced pH dependence of Pi recovery, with slower recovery at lower pH ($\tau_{\mathrm{Pi}}$: 19 ± 6 s at pH 7.3, 25 ± 7 s at pH 7.0, 32 ± 11 s at pH 6.8, and 46 ± 17 s at pH 6.6). Pi recovery is slowed with aging under acidic conditions, with little or no effect observed at neutral or alkaline pH. These results provide new insights into skeletal muscle metabolic heterogeneity, reflecting how different myofiber microenvironments modulate ATP synthesis. **Conclusions:** By overcoming the constraints of the single-pool model, the proposed multi-pool framework uncovers pH-dependent ATP synthesis and provides direct evidence of pronounced metabolic heterogeneity in skeletal muscle during exercise and recovery.

## 1. Introduction

ATP synthesis is a primary function of mitochondria, and its impairment has been linked to exercise intolerance in aging [1,2,3] and a variety of diseases such as mitochondrial myopathies [4], sarcopenia [5], insulin resistance [6], Type 2 diabetes (T2D) [7], Parkinson’s disease [8], heart failure [9], and obesity-related metabolic dysfunction [10]. For decades, dynamic ^31^P magnetic resonance spectroscopy (MRS) has been used to assess in vivo ATP synthesis, with the post-exercise PCr resynthesis rate serving as a reliable index of mitochondrial oxidative capacity [11,12,13,14,15]. However, this commonly used approach models PCr recovery as a mono-exponential process, implicitly assuming metabolic homogeneity of the active muscle—an assumption that conflicts with the well-documented heterogeneity of muscle fiber composition and the associated metabolic responses [13,14,15,16,17,18,19,20]. Muscle metabolic heterogeneity is further supported by evidence including regional differences in PCr depletion observed with localized ^31^P MRS, end-exercise pH-dependent variability in PCr recovery rates, and inhibition of mitochondrial oxidative phosphorylation under intracellular acidosis [11,21,22,23]. Complementing these findings, cell-based studies demonstrate that pH modulates glycolytic flux and affects mitochondrial ATP resynthesis [24,25,26]. Given the well-established roles of pH in skeletal muscle energy metabolism, it is desirable to explicitly integrate pH and ATP synthesis into a coherent framework.

Therefore, the present study aims to develop an approach that enables pH-resolved assessment of ATP synthesis using non-localized dynamic ^31^P MRS, leveraging its high signal-to-noise ratio and temporal resolution. Integrating pH into the assessment of ATP synthesis is both metabolically justified and technically feasible. From a metabolic perspective, pH acts not only as a key regulator of ATP synthesis but also as an integrative index of muscle metabolic state, reflecting proton release and uptake across multiple pathways and cellular compartments during exercise and recovery.

Notably, both pH and ATP synthesis can be probed through the inorganic phosphate (Pi) ^31^P MRS signal. First, the chemical shift of Pi directly reflects intracellular pH (HPO_4_^2−^ + H^+^ ⇌ H_2_PO_4_^−^, pKa 6.73). Second, Pi serves as a substrate for mitochondrial ATP synthesis (Pi + ADP → ATP). Together, these properties position Pi at the metabolic core of the cellular energetic apparatus, and the Pi ^31^P signal as a suitable dual marker for pH-resolved ATP synthesis.

To achieve the aim of pH-resolved ATP synthesis experimentally, a submaximal exercise regimen is used to induce a substantial increase in Pi signal intensity (approximately 5-fold relative to rest) as well as broader dispersion of the Pi resonance (~1 ppm). Under these conditions, the lineshape of the Pi signal can be segmented into four pre-defined pH regions spanning alkaline, neutral, mild acidic, and moderate acidic ranges, allowing continuous monitoring of Pi dynamics in each pH region as a function of recovery time. The resulting four-pool model links pH to ATP synthesis and therefore captures metabolic heterogeneity associated with myofiber composition within the activated muscle.

## 2. Materials and Methods

### 2.1. Human Subjects

The protocol was approved by the Institutional Review Board at the University of Texas Southwestern Medical Center. Prior to the MR spectroscopy (MRS) study, written informed consent was obtained from all participants. The study involved 5 individuals (4 F/1 M, age 45–79 yrs, Table 1) without known neuromuscular and skeletomuscular diseases. Participants were instructed to avoid any moderate or intense physical activity for 24 h before the study and to remain at rest for 20 min prior to the scan. All subjects tolerated the MRI scan and in-magnet exercise without issues.

### 2.2. MRS Protocol

#### 2.2.1. MRI Scanner, RF Coil, Exercise Device, and Subject Positioning

All participants were positioned feet-first and supine in the MRI scanner (7T Achieva; Philips Healthcare, Cleveland, OH, USA), with the calf muscle of the non-dominant leg centered within the RF coil (Philips Healthcare). The RF coil used was a half-cylinder-shaped, partial volume, double-tuned ^1^H/^31^P quadrature TR coil, consisting of two tilted, partially overlapping 10-cm loops. This coil was mounted on a solid base board of an ergometer that could be firmly attached to the MRI scanner table. Leg position was secured using Velcro straps, with a thick cushioning pad placed under the straps for added comfort. Participants were instructed to remain still and avoid muscle contractions, except during the supervised in-magnet exercise.

The ergometer was a custom-built, 7T-compatible single-legged exercise device equipped with a pedal-driven pulley system, allowing participants to perform exercises at a prescribed workload. To ensure proper isolation of the exercised muscle and maintain stability, cushioned straps were applied around the ankle and knee of the exercising leg, with an additional strap placed around the pelvis to enhance body stability.

To assess test–retest reproducibility, each participant was scanned twice following the same scanning protocol, with #1 and #2 scans separated by a 10-min interval within the same session, without the participant leaving the scanner table. For imaging, axial, and sagittal T2-weighted turbo spin echo images were acquired for shimming purposes. Second-order, ^1^H-based automatic volume shimming was applied before the ^31^P spectral acquisitions. PCr full linewidth at half height was <30 Hz for all subjects.

#### 2.2.2. Dynamic ^31^P MRS with Plantar Flexion

To measure myocellular ATP synthesis in skeletal muscle, real-time dynamic ^31^P MR spectra were acquired from the exercising muscle. The exercise protocol consisted of 2 min of single-legged rhythmic plantar flexion, with the knee kept straight, performed in the supine position. During this exercise, the participant’s foot pressed against a pedal with a constant workload, personalized to 20% of the subject’s body weight (BW), which was identical for both scans, and was similar across participants (14.8 ± 1.3 kg).

The in-magnet exercise consisted of 60 repetitions, each lasting 2 s–1 s of active plantarflexion involving concentric contraction against the 20% BW load, followed by 1 s of eccentric contraction under the same load to return to the initial position. Participants followed this exercise cadence given by the MRI operator through a telecom speaker, while a staff member in the scanner room monitored protocol compliance. The dynamic ^31^P MR spectra were acquired with a temporal resolution of 2 s, synchronized with the rate of the plantar flexion exercise. There was a total of 200 dynamic scans, including 10 at rest, 60 during exercise, and 130 during post-exercise recovery. The steady-state signals obtained from resting muscle (reached after 4 dynamic scans) served as an intensity reference, allowing for the assessment of signal dynamics during muscle contraction and subsequent recovery. Other standard ^31^P MRS parameters included excitation pulse flip angle 55°, 4k sampling points, zero-filled to 8k before Fourier transformation, and a transmitter carrier frequency offset of 50 Hz downfield from the α-ATP signal.

To characterize metabolite concentrations, an additional full relaxed ^31^P MR spectrum was obtained using long TR = 25 s and 16 acquisitions.

### 2.3. ^31^P MRS Data Analysis

All dynamic ^31^P MR spectra were aligned to the PCr signal (set to 0 ppm) to correct potential Bo variations during exercise and recovery. Gaussian apodization was applied to each free induction decay (FID) before Fourier transformation using the scanner software (SpectroView version R5.7; Philips Healthcare).

Frequency-domain ^31^P spectra were baseline-corrected, and signal intensities for Pi, PCr, and ATP were measured by integral (peak area under curve) and normalized by the corresponding steady-state values at rest, yielding relative magnetizations for each metabolite. The time courses of relative magnetizations and pH during recovery were fit to a mono-exponential equation to evaluate the recovery time constant τ using a home-built data analysis program written in MATLAB (version R2021b, The MathWorks, Inc., Natick, MA, USA), as described previously [8].

Specifically, the recovery time constant τ_PCr_, an index of skeletal muscle mitochondrial oxidative phosphorylation function, was evaluated by fitting the post-exercise PCr signals to the following equation according to the single-pool model:

(1) $$PCr(t)=PCr{|}_{\infty }-\Delta PCr\cdot e^{-t/\tau_{PCr}}$$

where PCr|_∞_ is the [PCr] after full recovery, ΔPCr = PCr|_∞_ − PCr|_ex_, in which PCr|_ex_ is the end-exercise [PCr].

Similarly, the Pi recovery time constant τ_Pi_, a direct index of de novo ATP synthesis Pi + ADP ⟶ ATP, was evaluated by the following equation:

(2) $$Pi(t)=Pi{|}_{\infty }+\Delta Pi\cdot e^{-t/\tau_{Pi}}$$

where Pi|_∞_ is the [Pi] after full recovery, ΔPi = Pi|_ex_ − Pi|_∞_, in which Pi|_ex_ is the end-exercise [Pi].

The pH was evaluated by the chemical shift (δ) of Pi signal relative to PCr using the following formula:

(3) $$pH=pKa+{log}_{10\;}\frac{\delta_{Pi}-\delta_{a}}{\delta_{b}-\delta_{Pi}}$$

where the H_2_PO_4_^−^ ↔ H^+^ + HPO_4_^2−^ deprotonation constant pKa = 6.73, and the ^31^P limiting shifts δ_a_ = 3.275 ppm (for acidic protonated species H_2_PO_4_^−^) and δ_b_ = 5.685 ppm (for basic deprotonated species HPO_4_^2−^) were used in the data analysis. To account for the asymmetric appearance of the Pi signal—particularly during exercise and early recovery—the Pi chemical shift used for pH evaluation was calculated as a lineshape magnitude-weighted δ, according to the following formula:

(4) $$\delta_{w}=\frac{\sum_{i}\;y_{i}\;\delta_{i}}{\sum_{i}\;\delta_{i}}$$

in which *y_i_* and δ*_i_* represent the signal magnitude and chemical shift at each data point i, respectively, and Σ denotes the summation over all data points within the Pi’s lineshape.

Based on the 1-to-1 relationship between δ and pH (Equation (3)), the multi-pool model is constructed by partitioning the lineshape coverage of the Pi signal into multiple consecutive δ-bins, with each pH pool defined as pHc ± ΔpH, where pHc denotes the central pH and ΔpH denotes the width of the pH pool. Considering the presence of four myofiber subtypes identified by biopsy (Type I, IIA, and IIX, plus a hybrid IIAX in humans, or IIB in animals) and the physiologically relevant pH range from alkaline (pH 7.5) to acidic (pH 6.5), as expected for submaximal exercise under normal physiology, a four-pool model was employed in the current study comprising the following pH pools: (a) pH 7.4 ± 0.2; (b) pH 7.0 ± 0.1; (c) pH 6.8 ± 0.1; and (d) pH 6.6 ± 0.1. Using this partition model, four distinct pool-specific Pi dynamic components were derived from the Pi lineshape, each characterized by its own recovery time constant (τ_Pi_). Similar to the analysis of the total Pi signal in the single-pool model, the dynamics of the pool-specific magnitude-weighted pH (denoted as pHw, to distinguish it from pHc) were also obtained from the pool-specific weighted chemical shift (δ), calculated according to Equation (4).

For the study group, the τ_Pi_ test–retest reproducibility was evaluated using the coefficient of variation (CV) derived from two independent scans acquired during the same session. The CV was calculated as the standard deviation divided by the mean and expressed as a percentage.

Muscle metabolite concentrations were quantified from the fully relaxed ^31^P MR spectrum acquired at the resting state. The concentration of each metabolite was determined from the area of the corresponding ^31^P resonance, obtained by Gaussian lineshape fitting, with γ-ATP used as an internal reference (set to 8.2 mM in skeletal muscle).

### 2.4. Statistical Analysis

All data were presented as mean ± standard deviation. Statistical significance was defined by *p* < 0.05. Given the small sample size (n = 5), linearity for correlation between two variables was evaluated by the Spearman test using the internal function *corr* in Matlab. For the 4-pool pH model, the difference in τ_Pi_ between two adjacent pools was evaluated by 1-tail type test (at the 95% confidence level).

## 3. Results

### 3.1. Dynamic ^31^P MRS and Analysis by Single-Pool Model

**Figure 1:**
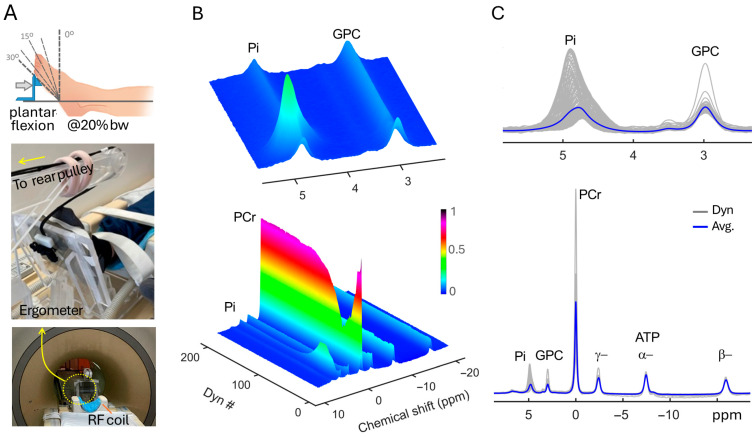
Group-summed dynamic 7T ^31^P MR spectra acquired from calf muscle at rest and during a 2-min plantar flexion exercise and the subsequent recovery. (**A**) Illustration of plantar flexion, and photos showing the setup of the exercise device in the MRI scanner. (**B**) Three-dimensional presentation of 200 sequential dynamic spectra. (**C**) Overlay of all 200 dynamic spectra. Note the broad, asymmetric Pi lineshape profile due to exercise-induced pH-dependent chemical shift dispersion, in contrast to the narrower, symmetric GPC resonance, which is insensitive to pH changes.

**Figure 2:**
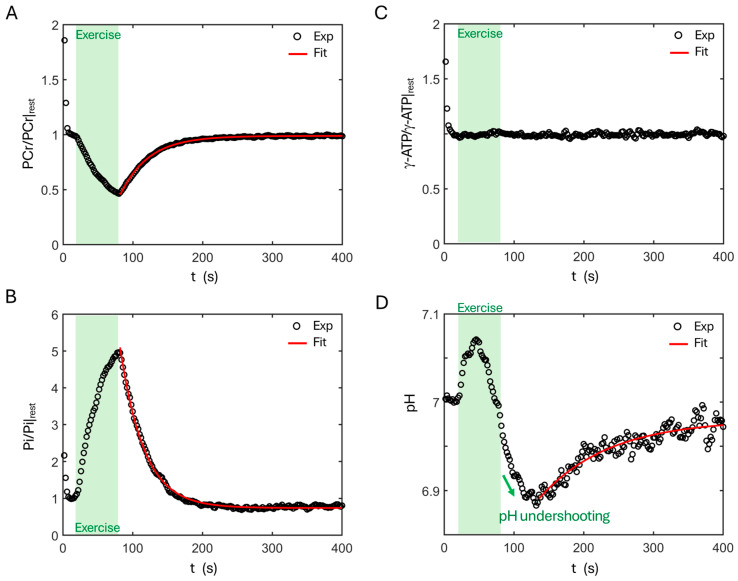
Time courses of PCr (**A**), Pi (**B**), γ-ATP (**C**), and pH (**D**) dynamics during exercise and recovery relative to the resting state.

Figure 1 presents a full set, group-summed dynamic ^31^P MR spectra acquired from calf muscle, showing rapid PCr decline and Pi rise during exercise, followed by a more gradual recovery phase in which PCr and Pi return toward their resting levels. The recovery of PCr and Pi appears to follow a mono-exponential process, with τ_Pi_ < τ_PCr_ (33.3 s vs. 40.2 s, Figure 2A,B). ATP signals remain nearly invariant throughout the exercise and recovery periods (Figure 2C).

In addition to a change in signal intensity, the Pi resonance also exhibits chemical shift change and asymmetric line broadening in response to exercise and recovery. The pH dynamics shows a “heartbeat-like” pattern, with an initial rise (alkalization) at the onset of exercise followed by a gradual decrease during the late phase of contraction; this trend persists for approximately 50 s after exercise (“pH undershoot”) before pH slowly returns toward its resting level over the remaining recovery period (τ_pH_ = 109 s, Figure 2D).

### 3.2. Correlation Between Pi, PCr and pH

**Figure 3:**
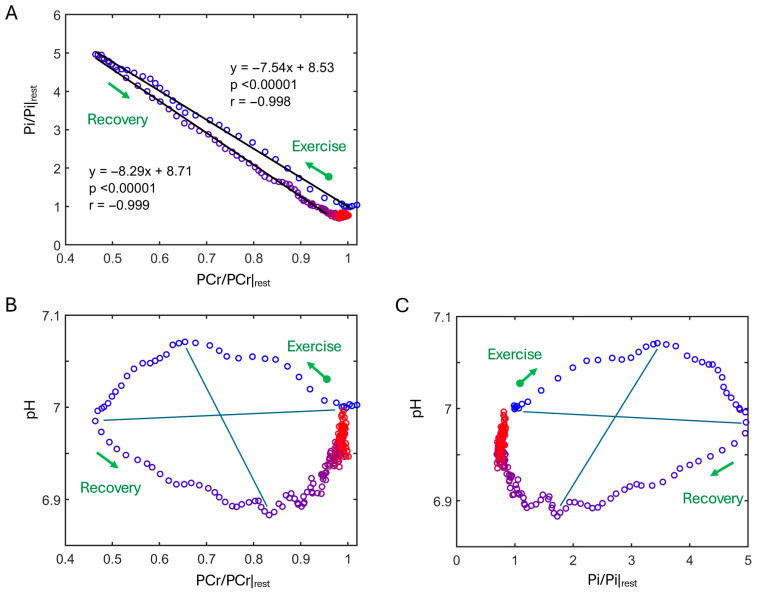
Interplay among Pi, PCr, and pH during exercise and recovery. (**A**) Pi vs. PCr, (**B**) pH vs. PCr, and (**C**) pH vs. Pi. The color gradient from blue to red represents the temporal transition rest → exercise → recovery. For O-shaped trajectories in (**B**,**C**), the four quadrants indicate phased dominance of distinct metabolic pathways over time: Quadrant I, CK-mediated PCr depletion with mild alkalization and Pi accumulation; II, anaerobic ATP synthesis from glycogen breakdown driving acidification with lactate buildup; III, lactate-fueled mitochondrial ATP production replenishing PCr with further acidification; IV, ATP synthesis surplus to PCr repletion, supporting glycogen resynthesis.

There is a linear correlation between Pi and PCr dynamics; however, the slope differs between the exercise and recovery phases (Figure 3A). Specifically, the data shows that more Pi is consumed to restore PCr during recovery than is released when PCr is depleted during exercise.

In contrast to this linear relationship between Pi and PCr, O-shaped trajectories are observed for the correlations between pH and PCr dynamics (counterclockwise, Figure 3B) and between pH and Pi dynamics (clockwise, Figure 3C). These trajectories are characterized by three turning points occurring in sequence: first, maximal alkalization (pH_max_ = 7.07) at 35% PCr depletion and a 3.5-fold rise in Pi; next, maximal PCr depletion (52%) with a 4.9-fold rise in Pi; and finally, maximal acidification (pH_min_ = 6.88) at 84% PCr recovery and a 1.65-fold rise in Pi relative to resting levels.

### 3.3. Four-Pool Model

**Figure 4:**
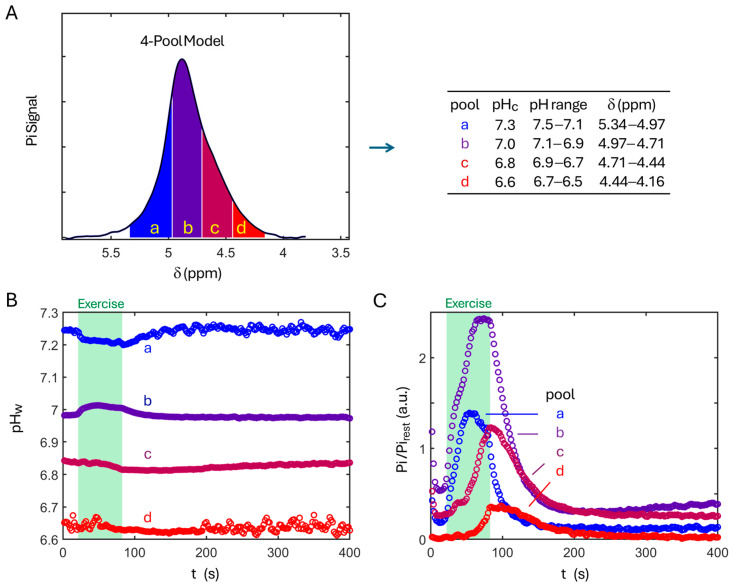
Multi-pool pH model based on δ-partition. (**A**) Scheme showing the partition of Pi lineshape into four δ-bins corresponding to four pH-pools from a to d. Dynamics of the weighted pH (**B**), and the corresponding Pi intensity (**C**), in response to exercise and recovery according to the 4-pool model.

As shown in Figure 4A, the four-pool model is based on partitioning the effective Pi signal range (5.34–4.16 ppm) into four distinct δ-bins, with each δ-bin corresponding to a specific pH range centered at pH_c_: (a) pH 7.3 ± 0.2, (b) pH 7.0 ± 0.1, (c) pH 6.8 ± 0.1, and (d) pH 6.6 ± 0.1.

Building on this partition framework, Figure 4B,C illustrate the dynamics of the pool-specific Pi signal intensity and the corresponding magnitude-weighted pH (pHw). Across all four pools, the pHw values remain largely stable during exercise and recovery (Figure 4B), particularly in the most acidic pool (pool-d). Within each pool, the averaged pHw variations are approximately 5–10-fold smaller than the pool’s predefined pH width and about 3.5-fold smaller than that evaluated using the single-pool model (Figure 4B vs. Figure 2D). In addition to this relative stability, the post-exercise pHw recovery is monotonic and faster in the alkaline pool than in the neutral and acidic pools (pool-a vs. pool-b and -c).

### 3.4. Pi Rise in Response to Exercise

In contrast to the relative stability of pHw, pronounced changes are observed in the Pi signal magnitude during exercise (Figure 4C). At resting steady state, Pi is predominantly distributed in the neutral pH pool (pH 7.0), whereas the most acidic pool (pH 6.6) is virtually absent, as anticipated. During exercise, Pi increases across all pools, rising initially more rapidly in the alkaline and neutral pools than in the acidic pools. Around the midpoint of the exercise bout, Pi reaches a maximum and begins to decline in the alkaline pool, while it plateaus after reaching a maximum in the neutral pool. In contrast, during the late phase of exercise, Pi accumulation accelerates in the two acidic pools, with a marked delay at pH 6.6 relative to pH 6.8. At the end of exercise, the magnitude of Pi change (ΔPi) across the four pools follows the order: pool-b > pool-a ~ pool-c > pool-d.

### 3.5. pH-Resolved ATP Synthesis

**Figure 5:**
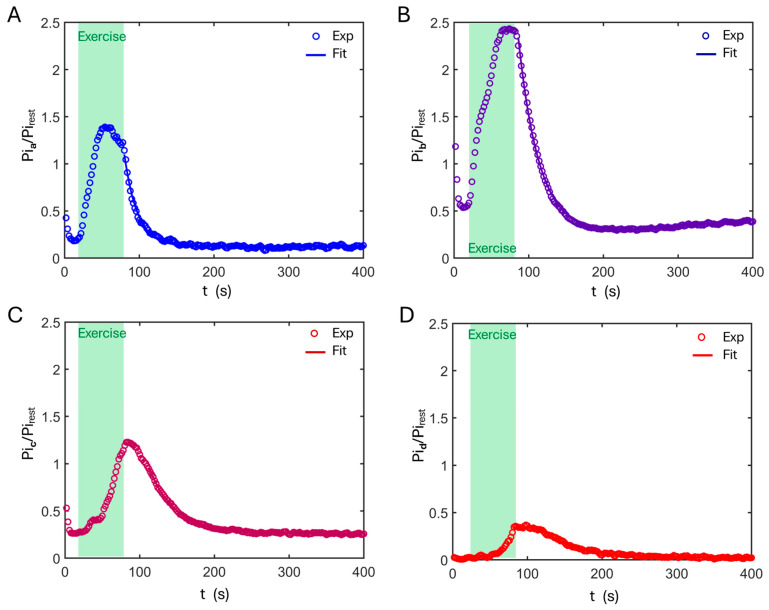
Time course and the mono-exponential fitting of post-exercise Pi recovery for the pH pools: pH 7.3 (**A**), pH 7.0 (**B**), pH 6.8 (**C**), and pH 6.6 (**D**), according to the 4-pool model.

Following the exercise phase, Pi progressively recovers toward its resting level in all pH pools. Pi recovery begins immediately in the alkaline and neutral pools, whereas a brief delay (~2–4 s) is observed at pH 6.8 and a more pronounced delay (~30 s) at pH 6.6 (Figure 4C). Despite these differences in onset time, Pi recovery in all pH pools is well described by a mono-exponential process (Figure 5).

**Figure 6:**
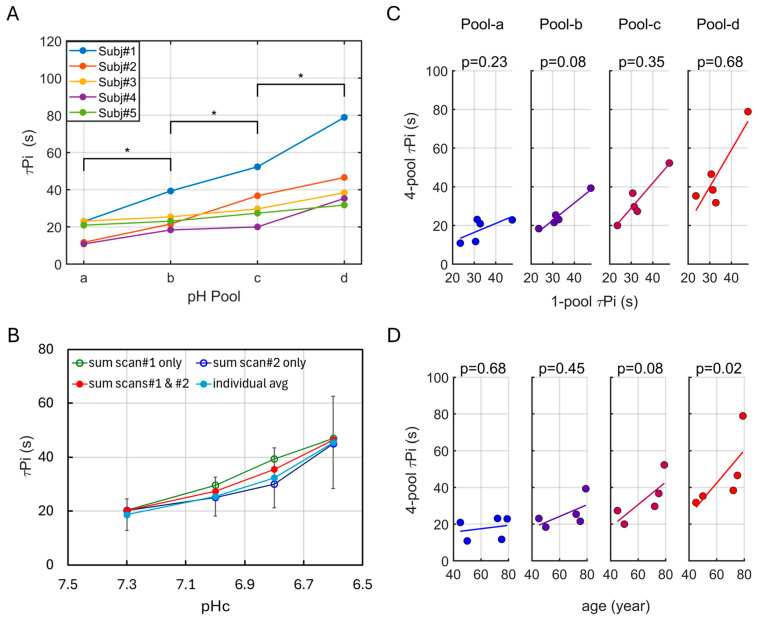
(**A**) Post-exercise Pi recovery time constants τ_Pi_ according to the 4-pool pH model, with * indicating *p* < 0.05 (n = 5). (**B**) τ_Pi_ versus pHc, with τ_Pi_ evaluated from spectra summed over all scans, only scan #1, scans #1 and #2, and only scan #2, respectively. (**C**) Linear correlation in τ_Pi_ between single-pool and 4-pool pH model (n = 5). (**D**) Linear correlation between age and τ_Pi_ by the 4-pool model (n = 5).

Assuming that mono-exponential Pi recovery after exercise is governed primarily by ATP resynthesis via the reaction Pi + ADP → ATP, pH-resolved ATP synthesis was quantified by fitting the Pi recovery curves for each pool. As summarized in Table 2, the fitted results show that ATP synthesis, assessed by the inverse Pi recovery time constant ($\tau_{Pi}^{-1}$), decreases with pH across all subjects (n = 5). Differences in τ_Pi_ between adjacent pH pools are statistically significant in the four-pool model (*p* < 0.05, Figure 6A). Notably, the τ_Pi_–pHc relationship is consistent between analyses based on summed spectra and those based on individual subjects (Figure 6B).

To further assess how the four-pool analysis relates to conventional approaches, Figure 6C compares τ_Pi_ values obtained from the single-pool and four-pool models. A relatively strong linear correlation is observed only for the most populated pool (pool-b), whereas the remaining pools show weaker relationships: pool-c exhibits a marginal correlation, and both the alkaline pool-a and acidic pool-d display poor correlation.

### 3.6. Age Dependence of τ_Pi_

Figure 6D shows the relationship between τ_Pi_ and age. An age-related increase in τ_Pi_ is observed, but this trend is pronounced primarily under acidic conditions, reaching statistical significance at pH 6.6 (*p* = 0.02) and showing a marginal effect at pH 6.8 (*p* = 0.08). In contrast, little to no age dependence is evident under neutral or alkaline pH conditions

Supplementary Figure S2 compares the linear correlation results (r, p) obtained with all participants (n = 5) and after exclusion of subject #1 (n = 4) to evaluate the potential influence of this subject on the age-dependent findings. In both analyses, the age-related association with τ_Pi_ remains evident in the two acidic pH pools, with no significant relationship observed in the alkaline or neutral pools.

### 3.7. Test–Retest Reproducibility of τ_Pi_ Measurements

For group-summed spectra, the coefficient of variation (CV) of τ_Pi_ across pH pools ranges from 0.06% to 19.1%, with a mean value of 12.4%. This variability compares with CVs of 7.3% for τ_PCr_ and 10.7% for τ_Pi_ estimated using the single-pool model. Under neutral and acidic conditions, τ_Pi_ values are shorter in the second scan than in the first scan (Figure 6B and Table 2).

### 3.8. Metabolite Concentration at Resting State

At rest, using γ-ATP (8.2 mM) as a reference, the group-averaged muscle metabolite concentrations are: PCr 37.4 ± 1.4 mM, Pi 4.2 ± 0.7 mM, PME 0.8 ± 0.1 mM, and PDE 5.1 ± 1.9 mM. The metabolite ratios are: PCr/Pi: 9.1 ± 1.8, PCr/γ-ATP: 4.6 ± 0.2, and γ-ATP/Pi: 2.0 ± 0.4. Gaussian lineshape deconvolution of resting calf muscle spectra reveals a small Pi component approximately 0.4 ppm downfield from the intracellular Pi resonance, although robust quantification of this peak in dynamic spectra is challenging, This Pi component corresponds to a tissue environment of approximately pH 7.4 and accounts for 14 ± 5% of the total Pi signal.

## 4. Discussion

Using real-time ^31^P MRS at high temporal resolution (2 s), this study characterized the dynamic changes in intracellular pH, Pi, and PCr, as well as their interplay during exercise and recovery. To better address metabolic heterogeneity associated with myofiber composition in skeletal muscle, we proposed a new analytical framework—pH-resolved ATP synthesis—that enables quantitative determination of ATP synthesis under distinct pH conditions. This development advances in vivo ^31^P MRS toward mechanistic assessment of metabolic regulation, moving beyond conventional quantification of metabolite concentrations and ATP fluxes.

### 4.1. Major Findings from the Single-Pool Model

ATP synthesis analysis by dynamic ^31^P MRS has long been based on post-exercise PCr recovery, which is often modeled as a mono-exponential process to derive the recovery time constant of PCr, an index of mitochondrial oxidative phosphorylation capacity that is coupled with cellular creatine kininase activity. The current study demonstrated that 7T ^31^P MRS permits Pi-based ATP synthesis analysis at the same temporal resolution as PCr. Furthermore, incorporating PCr dynamics with concurrent Pi and pH time courses provides a more comprehensive view of the metabolic coupling among these metabolites. The major findings derived from the single-pool model include: (1) Depletion of intracellular PCr results in proportional Pi accumulation during exercise. (2) pH dynamics exhibits a “heartbeat-like” pattern in response to exercise and recovery, characterized by an initial alkalization, followed by a neutralizing pH drop, a post-exercise acidic pH undershoot, and finally a slow recovery toward the resting state. (3) Metabolite recovery time constants follow τ_pH_ > τ_PCr_ > τ_Pi_.

### 4.2. Linearity in PCr and Pi Dynamics

During exercise, the rise in Pi is proportional to the depletion of PCr (Figure 2A). At the end of exercise, the measured PCr depletion (ΔPCr ≈ 50%, 18.5 mM) agrees well with the increase in Pi (~5-fold; ΔPi ≈ 17.8 mM). This suggests that there is an approximately 1:1 relationship between ΔPCr and ΔPi, likely reflecting a full visibility of Pi as PCr in cytosol during exercise. During early recovery, the linearity between PCr and Pi dynamics remains, but at a slightly increased slope, suggesting that more Pi disappeared than PCr recovered. While the exact reasons are not clear, one possibility may be that a small portion of Pi signals becomes dispersed into the spectral baseline due to continued acidification. However, other possibilities cannot be fully excluded, such as (1) an increased partial saturation effect at Pi due to potential T1 lengthening and/or T2 shortening after exercise (an NMR phenomenon); and (2) reduced Pi visibility resulting from increased Pi uptake into the densely packed mitochondrial matrix (~70% protein by mass), where macromolecular crowding may limit Pi diffusivity [27,28].

### 4.3. Pi Serves as a Dual Probe to Measure pH and ATP Synthesis

Despite being regarded as a “by-product” of ATP hydrolysis during energy production, Pi is an essential substrate for de novo ATP synthesis (Pi + ADP ⟶ ATP) and an easily accessible anion in maintaining in vivo acid–base equilibrium (H^+^ + HPO_4_^2−^ ↔ H_2_PO_4_^−^, pKa = 6.73). Evidently, Pi offers a distinct advantage over PCr as an endogenous dual ^31^P MRS probe for reporting pH-resolved ATP synthesis, as it reflects processes directly at the core of the cellular energetic machinery without concerns about spatial or temporal mismatches between these two measurements.

Regarding Pi detection, despite its relatively low muscle concentration (~4 mM at rest, which can rise rapidly with exercise), the ultrahigh field 7T provides sufficient spectral SNR, allowing reliable acquisition of Pi dynamics at a high temporal resolution of 2 s (Figure 1, Figure 2, Figure 3 and Figure 4, and Figure S1, Supplementary Materials). Beyond sensitivity and temporal resolution, Pi exhibits a large dynamic range in both signal intensity (~ 5-fold rise) and chemical shift (spanning over ~ 1 ppm) in response to exercise and recovery, a feature that empowers robust assessment of pH-resolved ATP synthesis (Figure 4, Figure 5 and Figure 6).

Consistent with prior studies [29,30], this study confirmed the presence of a small downfield Pi resonance, corresponding to a tissue environment at near pH 7.4. A similar Pi signal in the brain has also been observed previously, characterized by a long T1, and a metabolic inertness toward ATP synthesis, and therefore attributed to extracellular origins including interstitial fluid and blood [31,32,33], though a mitochondrial origin (pH 7.4–7.5) may also co-resonate in this chemical shift region (if fully visible by ^31^P MRS). Currently, there is a lack of spectral evidence demonstrating that this small downfield Pi is directly involved in ATP synthesis in skeletal muscle, in contrast to the cytosolic Pi component—the major, metabolically active Pi component [34].

Unlike spectra acquired at rest, robust quantification of this small downfield Pi resonance in dynamic spectra during muscle exercise and recovery is challenging. However, given its relatively alkaline pH, apparent metabolic inertness with respect to ATP synthesis, and physical origin from a comparatively small tissue compartment, this small downfield Pi component is not expected to substantially bias the corresponding τ_Pi_ estimate derived from either the single-pool or four-pool model. Importantly, the neutral and acidic Pi pools remain unaffected, as the downfield resonance virtually does not overlap with those spectral regions. Notably, in the present study, the post-exercise Pi signal in the alkaline pool increased approximately 5–7-fold relative to its resting level. Such a wide dynamic range would still permit reliable τ_Pi_ quantification, even if the downfield Pi component was considerably larger (e.g., in certain muscle disorders).

Given the much higher intramuscular concentration of PCr relative to Pi, and the absence of extracellular PCr contamination, one may ask whether PCr, like Pi, could also be used to assess pH-resolved ATP synthesis. Addressing this requires consideration of their differences in phosphate deprotonation behavior. Unlike Pi, the chemical shift of PCr is insensitive to pH change: its low pKa values (pKa_1_ < 1.7 and pKa_2_ ≈ 4.5) place its protonation equilibria well outside the physiological pH range between pH 6.5 and 7.5 [35]. This makes PCr unsuitable as a pH probe in vivo under normal physiology.

### 4.4. Major Findings from Pi-Based Four-Pool Model

By partitioning the Pi lineshape into four δ-bins corresponding to regions with distinct pH values, we examined pH-resolved ATP synthesis and gained deeper insight into the pH dependence of ATP energetics—a feature of metabolic heterogeneity in skeletal muscle that is not readily captured by the simplified single-pool model. The major findings derived from the four-pool model include: (1) the post-exercise Pi recovery time constant (τ_Pi_) increases as pool pH decreases; (2) acidic pH delays the onset of post-exercise Pi recovery; and (3) the age-dependent increase in τ_Pi_ is more pronounced at lower pH. Together, these findings indicate that acidic intracellular conditions inhibit ATP synthesis and that older individuals may be more susceptible to this pH-related inhibition.

Collectively, these findings from the four-pool model indicate that the PCr recovery time constant (τ_PCr_), as derived from the conventional single-pool model, should not be interpreted as a fixed measure of maximal energetic capacity for a given subject. This view is supported by prior studies demonstrating that τ_PCr_ varies with factors such as exercise intensity and end-exercise pH [22,36]. Such variability is rooted in the heterogeneous myofiber composition of skeletal muscle, which contributes to differences in PCr depletion, pH, and Pi recovery [11], even when end-exercise pH is constrained within the recommended range of 6.9–7.0 in the single-pool model [13].

Therefore, the newly proposed four-pool model complements the conventional single-pool model in illustrating muscle energy metabolism: the single-pool model provides a simple overview of the metabolic features and the coupling among PCr, Pi, and pH dynamics, whereas the four-pool model reveals pH-dependent heterogeneity in Pi recovery and ATP synthesis, offering more insights into pH regulation of muscle energetics that is consistent with muscle fiber composition.

### 4.5. Metabolic Heterogeneity: PCr, pH and Fiber-Types

Human biopsy and single-fiber studies have found significant variability in PCr concentration between and within fiber types, both at rest and after exercise [17,18,19,37,38]. For example, a study reported 20% greater resting PCr in fast, type II compared with slow, type I fibers in human skeletal muscle [17]. During exercise, PCr content is depleted more in type II fibers than in slow, type I fibers (e.g., ~62% vs. ~46% after 10 s of maximal work) [18], and a larger Pi rise and a deeper pH acidification in type II fibers compared to type I fibers under similar energy demand [19]. However, this metabolic heterogeneity is averaged out in the single pool model.

### 4.6. Remarks on the Four-Pool pH Model: Fiber-Typing Considerations

In this study, we adopted the four-pool pH model to provide a physiological reference in alignment with myofiber–type classifications established by biopsy studies. Although muscle fibers are often broadly categorized as type I (slow-twitch, oxidative) and type II (fast-twitch, glycolytic), biopsy data have identified multiple type II subtypes and hybrid fibers [39]. For example, based on myosin heavy chain isoforms and high-energy phosphate content, Sant’Ana Pereira et al. classified human muscle fibers into four types: type I, type IIA, type IIX, and hybrid type IIAX [40]. These fibers differ in oxidative and glycolytic capacities, and consequently, in their responses to functional demands and in their handling of Pi and pH [40,41,42,43].

In view of these metabolically discriminative features, muscle fibers with higher oxidative capacity are expected to maintain a more neutral to alkaline intracellular pH, whereas more glycolytic fibers are prone to a greater H^+^ accumulation and acidification. Of course, this correspondence does not imply a one-to-one mapping between individual fibers and specific pH pools, since distinct fiber types may overlap in metabolic response, and together, they may form a metabolic continuum [43]. Nevertheless, the multi-pool model enables systematic investigation of fiber-type–related changes and metabolic responses to factors such as neuromuscular activity, mechanical loading, and hormonal status—each known, through sustained or repeated exposure, to drive adaptive shifts in muscle fiber phenotype. For example, endurance exercise and chronic low-frequency stimulation promote fast-to-slow transitions [44], whereas reduced activity or mechanical unloading (e.g., disuse or microgravity) favors slow-to-fast shifts [45]; thyroid hormone status further modulates fiber phenotype, with hyperthyroidism promoting slow-to-fast conversion and hypothyroidism delaying fast-twitch characteristics [46]. Moreover, this framework can be applied to study muscles with selective fiber-type loss or functional deficits arising from disease, injury, or aging. In this context, the multi-pool pH framework offers intrinsic flexibility by allowing pH pool number and boundaries to be adapted to training- or disease-related phenotypic changes, thereby providing a clear advantage over single-pool approaches that may mask clinically relevant metabolic heterogeneity.

### 4.7. Acidic pH Undershoot and the Underlying Mechanisms

The single-pool model in this 7T study clearly reveals a transient pH undershoot, characterized by a continued decline in pH of approximately 0.1 units lasting ~50 s after the cessation of exercise (Figure 2D). This acidic pH undershoot is consistent with an early study by Takahashi et al., who reported that the minimum pH occurred at 45–55 s from the end of exercise in runners and controls in thigh muscles performing light and moderate knee extension exercise [41]. With a temporal resolution of 8 s, they also reported increased pH undershoot lasting for 57 s in runners and 94 s in controls after severe exercise, and 75 s in runners and 116 s in controls in exhausting exercise [41]. Although such an acidic pH undershoot phenomenon has been reported previously, it was often difficult to resolve—or was poorly resolved—in earlier low-field studies because of limited SNR and temporal resolution (≥10 s) [47,48,49], compared with the 2 s resolution achieved here. Two mechanisms may contribute to this apparent pH undershoot. For example, metabolically, early recovery is dominated by PCr resynthesis, a process that releases protons and can transiently drive intracellular acidification. Furthermore, the finding of pH-dependent Pi recovery from the four-pool model also points to an NMR-related effect: Pi signal recovers more rapidly in the downfield (more alkaline) portion of the spectrum than in the upfield (more acidic) portion, resulting in an asymmetric recovery which shifts the magnitude-weighted Pi centroid toward the upfield side, and consequently leading to an apparent decrease in the measured pH according to Equation (3).

### 4.8. pH-Dependent ATP Synthesis

As demonstrated by the $\tau_{\mathrm{Pi}}$–pH relationships derived from Pi lineshape analysis (Figure 6), ATP synthesis slows markedly as intracellular pH decreases, and is fastest under alkaline conditions, with more than a twofold difference in $\tau_{\mathrm{Pi}}$ between the alkaline (pH 7.2) and strongly acidic (pH 6.6) pools. This pH dependence revealed by the four-pool model is consistent with the single-pool observation of slower PCr recovery at higher exercise intensities and lower end-exercise pH. In both cases, increased metabolic stress shifts the Pi population toward more acidic compartments, where ATP synthesis kinetics are intrinsically slower [23]. Importantly, this pH-dependent variation may also explain inter-subject differences in PCr recovery between endurance-trained runners and untrained controls: minimal differences at low exercise intensity versus pronounced differences at high intensity [50].

### 4.9. Perspective of Non-Invasive Metabolism-Based Fiber-Typing

Currently, muscle biopsy has been regarded as the gold standard for fiber-type determination because it provides direct microscopic identification of individual fibers and enables precise classification based on myosin heavy chain isoform expression, enzymatic activity, and structural features. Although different biopsy techniques allow detection of hybrid fibers at the single-fiber level, they are invasive and destructive, are subject to tissue damage and sampling bias, and cannot be performed repeatedly in the same muscle or subject. Consequently, biopsies are poorly suited for longitudinal studies and for investigations involving pediatric, elderly, or vulnerable clinical populations.

In contrast, ^31^P MRS is non-invasive and repeatable, enabling dynamic in vivo assessment of fiber-type–related metabolic responses and recovery kinetics under physiological and pathophysiological conditions. By resolving pH-dependent Pi signals and modeling multiple pH pools, ^31^P MRS provides a functional measure of metabolic heterogeneity within the activated muscle volume that may reflect underlying fiber-type composition.

Therefore, the multi-pool ^31^P MRS approach can complement biopsy-based fiber typing by providing a non-invasive, functional perspective on muscle metabolic heterogeneity. When interpreted with established histological findings, this framework enables a more comprehensive understanding of fiber-type-dependent energetics in vivo, while avoiding many of the practical and ethical constraints associated with invasive tissue sampling.

### 4.10. Limitations and Remarks on Future Direction

This study has several limitations. First, the group size is small (n = 5 subjects), though each subject was scanned twice to assess test–retest reproducibility. Future studies should include a large cohort to validate the results presented here. Second, the exercise regimen used was set to 20% BW to induce a large PCr depletion and consequently large Pi and pH dynamic responses. Further investigation is needed into how changes in workload and duration of exercise will influence the results. Finally, the cohort of subjects studied are relative healthy, despite being overweight (reflecting the adult population profile in the U.S.). Extending this pH-resolved approach to diverse clinical populations will be important for understanding its sensitivity to metabolic disease and to interventions such as exercise training, dietary modification, and other therapeutic strategies.

It is important to consider the technical limitations when interpreting pH-resolved ^31^P MRS data for fiber typing. (1) Spatial resolution is limited relative to histological methods due to constrains from RF coil sensitivity and coverage. (2) Temporal resolution may be reduced when scanners at lower magnetic field strengths are used. (3) Integrated physiological factors—including muscle recruitment patterns, regional perfusion and oxygen delivery, and differences in metabolite transport or buffering capacity—can contribute to pH heterogeneity independently of intrinsic fiber-type properties and should be accounted for when interpreting multi-pool pH models.

It is worth noting that, although slowed PCr recovery has been linked to declined muscle energetic function with aging [22,51,52], a recent systematic review and meta-analysis of ^31^P-MRS studies found substantial heterogeneity in recovery τ_PCr_ across healthy individuals and muscle groups. Specifically, the analysis concluded that in many healthy muscles, the overall PCr recovery rates are similar across age groups, with only modest or non-significant correlations with age in certain muscles, such as the forearm and lower leg [22]. Others have observed even greater fatigue resistance in older adults [53,54]. These divergent findings suggest that the mechanisms underlying age-related reductions in muscle energetic power and increased exercise intolerance remain incompletely understood [41]. Age-related impairments are strongly influenced by experimental conditions and task demands. Consistent with this view, our data suggests that age-related limitations in ATP synthesis are most pronounced under exercise conditions that induce substantial muscle acidification, whereas aging has minimal impact at neutral or alkaline pH (Figure 6). A sensitivity analysis further indicates that these findings are not driven by a single subject (Supplementary Figure S2). Nevertheless, given the limited sample size (n = 5), this aging-related pH dependence warrants validation in larger cohorts to better understand age-associated functional decline and to inform the development of effective interventional strategies.

## 5. Conclusions

In conclusion, this work highlights inorganic phosphate (Pi) as a valuable endogenous dual ^31^P MRS probe for simultaneously assessing intracellular pH and ATP synthesis during muscle recovery. By exploiting the strong pH dependence of the Pi chemical shift and its robust recovery dynamics, the proposed pH-resolved multi-pool framework reveals metabolic heterogeneity that is obscured by conventional single-pool analyses. This approach provides a physiologically grounded and technically practical means to interrogate mitochondrial function and its regulation under varying metabolic states, with potential utility in both basic physiology and clinical studies of muscle metabolism.

## Figures and Tables

**Table 1:** Subject demographic data.

						preEX#1	postEX#1	preEX#2	postEX#2
Subj	Sex	Age/yr	BW/kg	BMI	sPO2/%	HR/bpm	HR/bpm	HR/bpm	HR/bpm
#1	M	79	69.1	25.1	99	57	70	58	75
#2	F	75	82.7	27.1	100	63	83	60	83
#3	F	72	75.6	28.8	99	82	91	80	97
#4	F	50	78.0	30.5	99	57	79	57	87
#5	F	45	64.3	26.3	100	65	80	65	81
Avg		64.2	74.0	27.6	99.4	64.8	80.6	64.0	84.6
SD		13.9	6.5	1.9	0.5	9.2	6.8	8.5	7.3

**Table 2:** Post-exercise Pi recovery time constants τ (s) by 1- and 4-pool models.

4-pool	Sum ^1^ All	Sum ^2^ Scan#1	Sum ^3^ Scan#2	Individual ^4^ Sum #1 & #2	Individual ^5^ Scan #1	Individual ^6^ Scan #2
a	20.3	20.3	20.3	18.7 ± 5.8 (31%)	18.6 ± 7.6	17.8 ± 5.3
b	27.4	29.6	25.1	25.4 ± 7.3 (29%)	27.1 ± 11.0	23.7 ± 4.7
c	35.5	39.4	30.0	32.4 ± 11.1 (34%)	34.7 ± 18.5	30.5 ± 8.9
d	46.4	46.9	45.0	45.5 ± 17.2 (38%)	56.5 ± 44.3	45.4 ± 13.4
**1-pool**						
τ_Pi_	33.3	35.9	31.0	33.2 ± 8.8 (27%)	35.4 ± 13.2	30.7 ± 4.9
τ_PCr_	40.2	42.4	38.3	40.4 ± 9.5 (24%)	42.7 ± 13.8	38.4 ± 5.8

^1^ From group-summed spectra for both #1 and #2 scans. ^2^ From group-summed spectra for scan #1 only. ^3^ From group-summed spectra for scan #2 only. ^4^ Averaged over individual subjects on summed spectra for both #1 and #2 scans. ^5^ Averaged over individual subjects on spectra #1 only. ^6^ Averaged over individual subjects on spectra #2 only.

## Data Availability

Data in this study are available upon reasonable request to the corresponding author.

## Supplementary Materials

The following supporting information can be downloaded at: https://www.mdpi.com/article/10.3390/diagnostics16050744/s1, Figure S1: Dynamic 7T 31P MR spectra acquired from the calf muscle at rest, during a 1-min plantar-flexion exercise, and throughout subsequent recovery in individual subjects (n = 5) in repeated scans (#1 and #2). Blue traces represent the averaged spectrum, while gray traces show an overlay of 200 dynamic spectra acquired at a temporal resolution of 2 s.; Figure S2: Sensitivity analysis assessing the influence of a potentially deviating subject on the age-dependent correlation results. Linear correlation coefficients (r) and *p* values are shown for the full cohort (n = 5; A) and after exclusion of one subject (n = 4; B).

## Abbreviations

The following abbreviations are used in this manuscript:

ATP	Adenosine triphosphate
ADP	Adenosine diphosphate
Cr	Creatine
PCr	Phosphocreatine
Pi	Inorganic phosphate
GPC	Glycerophosphocholine
MRS	Magnetic Resonance Spectroscopy
PME	Phosphate monoester
PDE	Phosphate diester

## References

[1] Grevendonk L., Connell N.J., McCrum C., Fealy C.E., Bilet L., Bruls Y.M.H., Mevenkamp J., Schrauwen-Hinderling V.B., Jörgensen J.A., Moonen-Kornips E. (2021). Impact of aging and exercise on skeletal muscle mitochondrial capacity, energy metabolism, and physical function. Nat. Commun..

[2] Layec G., Trinity J.D., Hart C.R., Kim S.E., Groot H.J., Le Fur Y., Sorensen J.R., Jeong E.K., Richardson R.S. (2015). Impact of age on exercise-induced ATP supply during supramaximal plantar flexion in humans. Am. J. Physiol.-Regul. Integr. Comp. Physiol..

[3] Cefis M., Marcangeli V., Hammad R., Granet J., Leduc-Gaudet J.P., Gaudreau P., Trumpff C., Huang Q., Picard M., Aubertin-Leheudre M. (2025). Impact of physical activity on physical function, mitochondrial energetics, ROS production, and Ca(2+) handling across the adult lifespan in men. Cell Rep. Med..

[4] Tarnopolsky M.A., Raha S. (2005). Mitochondrial myopathies: Diagnosis, exercise intolerance, and treatment options. Med. Sci. Sports Exerc..

[5] Zhu Y., Zhou X., Zhu A., Xiong S., Xie J., Bai Z. (2023). Advances in exercise to alleviate sarcopenia in older adults by improving mitochondrial dysfunction. Front. Physiol..

[6] Kim J.A., Wei Y., Sowers J.R. (2008). Role of mitochondrial dysfunction in insulin resistance. Circ. Res..

[7] Pinti M.V., Fink G.K., Hathaway Q.A., Durr A.J., Kunovac A., Hollander J.M. (2019). Mitochondrial dysfunction in type 2 diabetes mellitus: An organ-based analysis. Am. J. Physiol.-Endocrinol. Metab..

[8] Ren J., Patel N., Johnson T., Querry R., Shearin S. (2025). Skeletal Muscle ^31^P Magnetic Resonance Spectroscopy Study of Patients with Parkinson’s Disease: Energy Metabolism and Exercise Performance. Diagnostics.

[9] Skow R.J., Sarma S., MacNamara J.P., Bartlett M.F., Wakeham D.J., Martin Z.T., Samels M., Nandadeva D., Brazile T.L., Ren J. (2024). Identifying the Mechanisms of a Peripherally Limited Exercise Phenotype in Patients with Heart Failure with Preserved Ejection Fraction. Circ. Heart Fail..

[10] Jun L., Tao Y.X., Geetha T., Babu J.R. (2024). Mitochondrial Adaptation in Skeletal Muscle: Impact of Obesity, Caloric Restriction, and Dietary Compounds. Curr. Nutr. Rep..

[11] Fiedler G., Schmid A., Goluch S., Schewzow K., Laistler E., Niess F., Unger E., Wolzt M., Mirzahosseini A., Kemp G.J. (2016). Skeletal muscle ATP synthesis and cellular H^+^ handling measured by localized ^31^P-MRS during exercise and recovery. Sci. Rep..

[12] Kemp G.J., Ahmad R.E., Nicolay K., Prompers J.J. (2014). Quantification of skeletal muscle mitochondrial function by 31P magnetic resonance spectroscopy techniques: A quantitative review. Acta Physiol..

[13] Meyerspeer M., Boesch C., Cameron D., Dezortová M., Forbes S.C., Heerschap A., Jeneson J.A.L., Kan H.E., Kent J., Layec G. (2020). ^31^P magnetic resonance spectroscopy in skeletal muscle: Experts’ consensus recommendations. NMR Biomed..

[14] Layec G., Gifford J.R., Trinity J.D., Hart C.R., Garten R.S., Park S.Y., Le Fur Y., Jeong E.K., Richardson R.S. (2016). Accuracy and precision of quantitative 31P-MRS measurements of human skeletal muscle mitochondrial function. Am. J. Physiol.-Endocrinol. Metab..

[15] Meyer R.A. (1988). A linear model of muscle respiration explains monoexponential phosphocreatine changes. Am. J. Physiol..

[16] Yoshida T. (2002). The rate of phosphocreatine hydrolysis and resynthesis in exercising muscle in humans using 31P-MRS. J. Physiol. Anthropol. Appl. Human Sci..

[17] Sahlin K., Söderlund K., Tonkonogi M., Hirakoba K. (1997). Phosphocreatine content in single fibers of human muscle after sustained submaximal exercise. Am. J. Physiol..

[18] Karatzaferi C., de Haan A., van Mechelen W., Sargeant A.J. (2001). Metabolism changes in single human fibers during brief maximal exercise. Exp. Physiol..

[19] Wernbom M., Aagaard P. (2020). Muscle fibre activation and fatigue with low-load blood flow restricted resistance exercise—An integrative physiology review. Acta Physiol..

[20] Kuznetsov A.V., Margreiter R., Hagenbuchner J., Ausserlechner M.J. (2025). Energy metabolism in different skeletal muscles and muscle fibers: Implications for injury and dietary supplementation. Pflug. Arch..

[21] Roussel M., Bendahan D., Mattei J.P., Le Fur Y., Cozzone P.J. (2000). ^31^P magnetic resonance spectroscopy study of phosphocreatine recovery kinetics in skeletal muscle: The issue of intersubject variability. Biochim. Biophys. Acta.

[22] Singh M., Jhajharia A., Pruthi R., Carmichael O.T. (2025). ^31^P-MRS-Measured Phosphocreatine Recovery Kinetics in Human Muscles in Health and Disease-A Systematic Review and Meta-Analysis. NMR Biomed..

[23] Jubrias S.A., Crowther G.J., Shankland E.G., Gronka R.K., Conley K.E. (2003). Acidosis inhibits oxidative phosphorylation in contracting human skeletal muscle in vivo. J. Physiol..

[24] Luzia L., Lao-Martil D., Savakis P., van Heerden J., van Riel N., Teusink B. (2022). pH dependencies of glycolytic enzymes of yeast under in vivo-like assay conditions. FEBS J..

[25] Kaminskas E. (1978). The pH-dependence of sugar-transport and glycolysis in cultured Ehrlich ascites-tumour cells. Biochem. J..

[26] Poburko D., Santo-Domingo J., Demaurex N. (2011). Dynamic regulation of the mitochondrial proton gradient during cytosolic calcium elevations. J. Biol. Chem..

[27] Scalettar B.A., Abney J.R., Hackenbrock C.R. (1991). Dynamics, structure, and function are coupled in the mitochondrial matrix. Proc. Natl. Acad. Sci. USA.

[28] Cooper G.M. (2000). The Cell: A Molecular Approach.

[29] van Oorschot J.W., Schmitz J.P., Webb A., Nicolay K., Jeneson J.A., Kan H.E. (2013). ^31^P MR spectroscopy and computational modeling identify a direct relation between Pi content of an alkaline compartment in resting muscle and phosphocreatine resynthesis kinetics in active muscle in humans. PLoS ONE.

[30] Valkovič L., Chmelík M., Ukropcová B., Heckmann T., Bogner W., Frollo I., Tschan H., Krebs M., Bachl N., Ukropec J. (2016). Skeletal muscle alkaline Pi pool is decreased in overweight-to-obese sedentary subjects and relates to mitochondrial capacity and phosphodiester content. Sci. Rep..

[31] Ren J., Sherry A.D., Malloy C.R. (2015). ^31^P-MRS of healthy human brain: ATP synthesis, metabolite concentrations, pH, and T1 relaxation times. NMR Biomed..

[32] Ren J., Yu F., Greenberg B.M. (2023). ATP line splitting in association with reduced intracellular magnesium and pH: A brain ^31^P MR spectroscopic imaging (MRSI) study of pediatric patients with myelin oligodendrocyte glycoprotein antibody-associated disorders (MOGADs). NMR Biomed..

[33] Ren J., Shang T., Sherry A.D., Malloy C.R. (2018). Unveiling a hidden ^31^P signal coresonating with extracellular inorganic phosphate by outer-volume-suppression and localized ^31^P MRS in the human brain at 7T. Magn. Reson. Med..

[34] Ren J., Sherry A.D., Malloy C.R. (2015). Amplification of the effects of magnetization exchange by ^31^P band inversion for measuring adenosine triphosphate synthesis rates in human skeletal muscle. Magn. Reson. Med..

[35] Chen L. (2017). Determination of thermodynamic acidity constants of phosphocreatine and its rate constants of hydrolysis reaction by pressure-assisted capillary zone electrophoresis. Biomed. Res..

[36] McMahon S., Jenkins D. (2002). Factors affecting the rate of phosphocreatine resynthesis following intense exercise. Sports Med..

[37] Söderlund K., Hultman E. (1991). ATP and phosphocreatine changes in single human muscle fibers after intense electrical stimulation. Am. J. Physiol..

[38] Tsintzas K., Williams C., Constantin-Teodosiu D., Hultman E., Boobis L., Clarys P., Greenhaff P. (2001). Phosphocreatine degradation in type I and type II muscle fibres during submaximal exercise in man: Effect of carbohydrate ingestion. J. Physiol..

[39] Pette D., Spamer C. (1986). Metabolic properties of muscle fibers. Fed. Proc..

[40] Sant’Ana Pereira J.A., Sargeant A.J., Rademaker A.C., de Haan A., van Mechelen W. (1996). Myosin heavy chain isoform expression and high energy phosphate content in human muscle fibres at rest and post-exercise. J. Physiol..

[41] Sundberg C.W., Hunter S.K., Trappe S.W., Smith C.S., Fitts R.H. (2018). Effects of elevated H^+^ and P_i_ on the contractile mechanics of skeletal muscle fibres from young and old men: Implications for muscle fatigue in humans. J. Physiol..

[42] Staron R.S., Pette D. (1993). The continuum of pure and hybrid myosin heavy chain-based fibre types in rat skeletal muscle. Histochemistry.

[43] Pette D., Staron R.S. (2000). Myosin isoforms, muscle fiber types, and transitions. Microsc. Res. Tech..

[44] Malisoux L., Francaux M., Theisen D. (2007). What Do Single-Fiber Studies Tell Us about Exercise Training?. Med. Sci. Sports Exerc..

[45] Ciciliot S., Rossi A.C., Dyar K.A., Blaauw B., Schiaffino S. (2013). Muscle type and fiber type specificity in muscle wasting. Int. J. Biochem. Cell Biol..

[46] Zhang D., Wang X., Li Y., Zhao L., Lu M., Yao X., Xia H., Wang Y.C., Liu M.F., Jiang J. (2014). Thyroid hormone regulates muscle fiber type conversion via miR-133a1. J. Cell Biol..

[47] Yoshida T., Watari H. (1992). Muscle metabolism during repeated exercise studied by 31P-MRS. Ann. Physiol. Anthropol..

[48] Raymer G.H., Green H.J., Ranney D.A., Marsh G.D., Thompson R.T. (2009). Muscle metabolism and acid-base status during exercise in forearm work-related myalgia measured with 31P-MRS. J. Appl. Physiol..

[49] Sullivan M.J., Saltin B., Negro-Vilar R., Duscha B.D., Charles H.C. (1994). Skeletal muscle pH assessed by biochemical and 31P-MRS methods during exercise and recovery in men. J. Appl. Physiol..

[50] Takahashi H., Inaki M., Fujimoto K., Katsuta S., Anno I., Niitsu M., Itai Y. (1995). Control of the rate of phosphocreatine resynthesis after exercise in trained and untrained human quadriceps muscles. Eur. J. Appl. Physiol. Occup. Physiol..

[51] Krumpolec P., Klepochová R., Just I., Tušek Jelenc M., Frollo I., Ukropec J., Ukropcová B., Trattnig S., Krššák M., Valkovič L. (2020). Multinuclear MRS at 7T Uncovers Exercise Driven Differences in Skeletal Muscle Energy Metabolism Between Young and Seniors. Front. Physiol..

[52] Giacona J.M., Afridi A., Bezan Petric U., Johnson T., Pastor J., Ren J., Sandon L., Malloy C., Pandey A., Shah A. (2024). Association between dietary phosphate intake and skeletal muscle energetics in adults without cardiovascular disease. J. Appl. Physiol..

[53] Christie A., Snook E.M., Kent-Braun J.A. (2011). Systematic review and meta-analysis of skeletal muscle fatigue in old age. Med. Sci. Sports Exerc..

[54] Rawson E.S. (2010). Enhanced fatigue resistance in older adults during repeated sets of intermittent contractions. J. Strength Cond. Res..

