# Supplementary material for: pH-Resolved ATP Synthesis in Skeletal Muscle: Concept, Implementation, and Assessment Using Dynamic 31P Magnetic Resonance Spectroscopy at 7T
Source: Diagnostics (Basel). 2026 Mar 2;16(5):744. doi: 10.3390/diagnostics16050744 (PMC12984577; doi:10.3390/diagnostics16050744)

Supplementary Materials

pH-Resolved ATP Synthesis in Skeletal Muscle: Concept, Implementation, and Assessment Using Dynamic ^{31}P Magnetic Resonance Spectroscopy at 7T

Figure S1. Dynamic 7T ^{31}P MR spectra acquired from the calf muscle at rest, during a 1-min plantar-flexion exercise, and throughout subsequent recovery in individual subjects (n = 5) in repeated scans (#1 and #2). Blue traces represent the averaged spectrum, while gray traces show an overlay of 200 dynamic spectra acquired at a temporal resolution of 2 s.

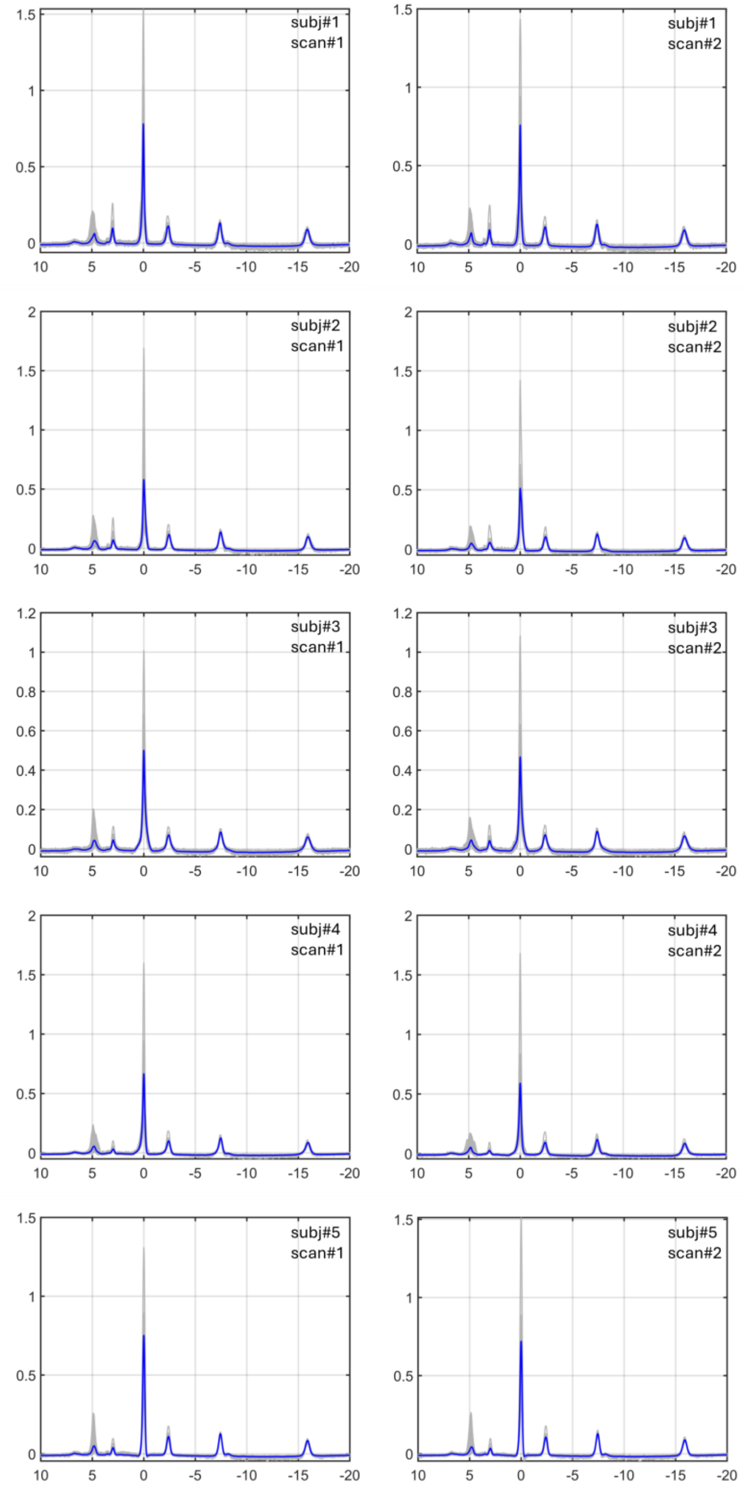

Figure S2. Sensitivity analysis assessing the influence of a potentially deviating subject on the age-dependent correlation results. Linear correlation coefficients (r) and p values are shown for the full cohort (n = 5; **A**) and after exclusion of one subject (n = 4; **B**).

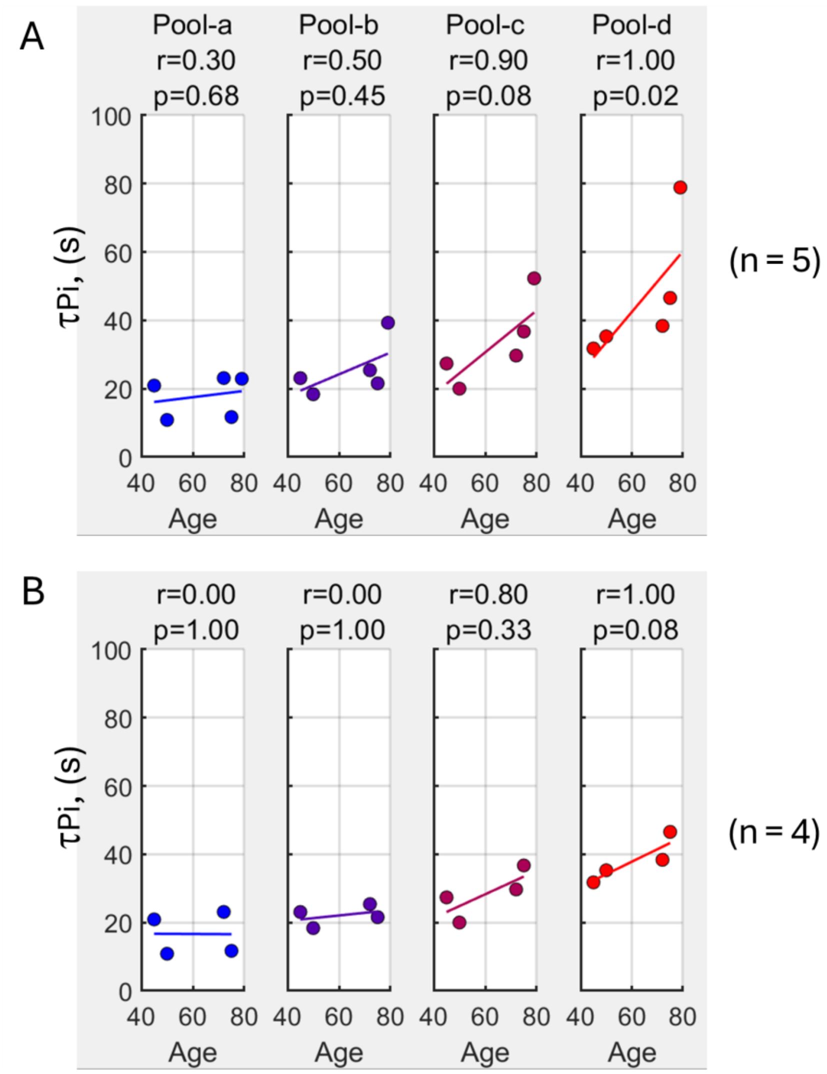

Supplement: Supplementary file 1 [file diagnostics-16-00744-s001.zip › diagnostics-4136844-supplementary.pdf]
